# Adenosine A_2B_ Receptor Antagonism Interferes with TGF-β Cellular Signaling Through SMAD2/-3 and p65-Nf-κB in Podocytes and Protects from Phenotypical Transformation in Experimental Diabetic Glomerulopathy

**DOI:** 10.3390/cells14120890

**Published:** 2025-06-12

**Authors:** Ignacio Arias, Claudia Jara, Pablo Mendoza-Soto, Yessica Nahuelpán, Claudio Cappelli, Carlos Oyarzún, Diego Carrillo-Beltrán, Claudia Quezada-Monrás, Angelo Torres-Arévalo, Rody San Martín

**Affiliations:** 1 Molecular Pathology Laboratory, Institute of Biochemistry and Microbiology, Science Faculty, Universidad Austral de Chile, Valdivia 5110566, Chile; iaact.cl@gmail.com (I.A.); claudia.jaracancino@gmail.com (C.J.); pa.men2a@gmail.com (P.M.-S.); nahuelpan.yessica@gmail.com (Y.N.); claudio.cappellileon@gmail.com (C.C.); carlosoyarzun@uach.cl (C.O.); 2 Tumor Biology Laboratory, Institute of Biochemistry and Microbiology, Science Faculty, Universidad Austral de Chile, Valdivia 5110566, Chile; diego.carrillo@uach.cl (D.C.-B.); claudiaquezada@uach.cl (C.Q.-M.); 3 Millennium Institute on Immunology and Immunotherapy, Universidad Austral de Chile, Valdivia 5110566, Chile; 4 Escuela de Medicina Veterinaria, Facultad de Medicina Veterinaria y Recursos Naturales, Sede Talca, Universidad Santo Tomás, Talca 3473620, Chile

**Keywords:** diabetic nephropathy, TGF-beta 1, adenosine receptors, glomerulosclerosis

## Abstract

Studies have emphasized alleviating fibrogenesis through interference with adenosine signaling in experimental diabetic nephropathy. We found that the in vivo antagonism of the adenosine A_2B_ receptor (A_2B_AR) using MRS1754 in diabetic rats impedes the diabetes-induced glomerular expression of the mesenchymal-like transformation markers Snail and α-SMA, while the loss of the epithelial podocyte-specific proteins nephrin and ZO-1 was prevented. Furthermore, the production of MCP-1, CCL3, TGF-β, and the transcript levels of inflammatory mediators was reduced by A_2B_AR antagonism. Using human podocytes in vitro, we demonstrated that A_2B_AR antagonism affected the TGF-β-induced activation of SMAD2/-3, as evidenced by the attenuated phosphorylation of SMAD2/-3 and decreased SMAD3 occupancy at target gene promoters following the MRS1754 treatment. Moreover, the non-canonical activation of p65-NF-κB, the primary inflammatory signaling pathway downstream of TGF-β, and the expression of Snail were also reduced by MRS1754. We conclude that an A_2B_AR blockade interferes with the pathogenic TGF-β signaling cascade responsible for the phenotypical transformation of podocytes, thereby alleviating diabetic glomerulopathy.

## 1. Introduction

Diabetic nephropathy (DN) is a complication of diabetes mellitus that affects 30–40% of diabetic people. DN manifests clinically with urinary protein leakage and progressive decline in the glomerular filtration rate. DN is characterized by increases in blood pressure and cardiovascular risk [1]. This complication of diabetes remains incurable and continues to be the major cause of terminal kidney disability worldwide [1].

Histological examinations of kidneys affected by DN demonstrate glomerulosclerosis and extensive tubulointerstitial fibrosis. This process progresses gradually, with a direct correlation between the degree of renal fibrosis and the loss of renal function [2]. Despite fibrosis being a common final pathway leading to organ loss in all chronic kidney diseases, there are currently no interventional options to reverse it [3,4]. Recent research highlights the role of phenotypic changes in renal resident cells that enable them to orchestrate fibrotic processes and generate myofibroblasts [5,6,7]. This process, occurring from the early stages of DN, is highly relevant because it perpetuates renal cell dysfunction, the remodeling of kidney tissue, and the loss of organ function. Podocytes are highly specialized epithelial cells anchored to the glomerular basement membrane via foot processes that are interdigitated by specialized junctional protein complexes known as the slit diaphragm. This array of cellular components comprises the urinary face of the glomerular filtration barrier [8]. During DN, podocytes undergo phenotypic changes resembling the epithelial-to-mesenchymal transition [8], a key event related to the loss of their specialized features required for efficient glomerular function [8,9,10]. Additionally, the mesenchymal-like transition of epithelial tubule cells has been linked to the acquisition of proinflammatory properties that promote fibrogenesis [6]. Transforming growth factor-beta 1 (TGF-β) is the main mediator of the extensive dedifferentiation of renal cells, proteinuria, and fibrosis [11,12]. TGF-β levels are induced in DN [13,14], playing a central role in the phenotypic transition of podocytes, podocyte loss, and increased glomerular permeability leading to proteinuria [15,16,17]. At the cellular level, canonical TGF-β signaling involves activating phosphorylation of the rSMADs, SMAD2 and -3, which complex with SMAD4 and translocate to the nucleus to control the expression of responsive genes and non-coding RNAs implicated in renal fibrosis [18,19]. SMAD3 has been recognized as playing a predominant role in the TGF-β-mediated dedifferentiation of podocytes [20] and their subsequent loss [21]. However, the pleiotropic effects of TGF-β in human physiology and pathology result from signaling crosstalk with various cellular pathways that coregulate its actions, such as NF-κB, MAPKs (Erk, p38MAPK, JNK), PI3K, the EGF receptor, Wnt, Notch, and JAK/STAT [11]. Evidence suggests that some non-canonical TGF-β signaling pathways, such as the hyperactivation of NF-κB, may play pivotal roles in renal fibrosis [22,23]. Furthermore, the activity of TGF-β signaling regulators can significantly influence TGF-β effects in renal cells, including podocytes [24]. Understanding how TGF-β regulators and intracellular signaling cascades operate in diabetic kidney disease may lead to new therapeutical strategies for renal fibrosis and proteinuria.

In recent years, the study of dysregulated adenosine homeostasis and the role of injurious signaling in DN has been the focus of research seeking new interventional options [4,7,25,26]. Progressive stages of DN were correlated with increased plasma adenosine levels in diabetic patients [27,28,29]. In experimental models of DN, the rise in adenosine levels parallels with histological and functional changes seen in incipient DN [25,29,30]. The effects of adenosine are mediated by four plasma membrane adenosine receptors, characterized by different ligand affinities [4]. In normal renal physiology, adenosine plays essential roles, such as regulating the glomerular filtration rate via tubuloglomerular feedback [4,31]. However, in diabetic kidneys, adenosine signaling properties can be altered due to increased adenosine levels and the induction of the lower-affinity adenosine A_2B_ receptor subtype (A_2B_AR), which occurs in both experimental and human DN [4,32]. The selective antagonism of A_2B_AR has alleviated glomerular alterations in experimental DN, offering protection against diabetes-induced proteinuria, the myofibroblastic transformation of cells, and the infiltration of immune cells [7,26,32,33]. Thus, this study investigated whether beneficial outcomes from A_2B_AR antagonism may affect the fibrogenic TGF-β axis to alleviate podocytopathy in experimental DN.

## 2. Materials and Methods

### 2.1. Experimental Diabetic Nephropathy

An experimental model of DN was generated using male Sprague Dawley rats weighing 200 grs (7 weeks old), purchased from Universidad de la Frontera de Chile’s vivarium, through a single i.v. dose of 65 mg/kg streptozotocin dissolved in buffer citrate (10 mM sodium citrate, 0.8% sodium chloride, pH of 4.5). The induction of diabetes was monitored by measuring the glucose levels in the blood from the tail at fasting (8 h). Animals with glycemia higher than 450 mg/dL were included in the study (corresponding to approximately 90%). After 4 weeks of diabetes induction, two groups were randomly generated and treated with MRS1754 (A_2B_AR antagonist, cat. 2752, Tocris, Minneapolis, MN, USA) at 0.5 mg/kg or a vehicle (PBS1×) administered intraperitoneally every 48 h. After the treatments, the animals were sacrificed using sodium thiopental (i.p. 100 mg/kg), and the kidneys were processed for the analyses. For this research, each animal was considered an experimental unit. The sample size was decided based on the number necessary to reach significant differences in the proteinuria among groups. The total number of animals used was 52. The animals were maintained under standard conditions, blinded to housing personal, kept in individual cages, and individualized through marks on the tail. The animal procedures were authorized by the Institutional Committee for Care and Use of Animals in Research at University Austral de Chile (Ref. 432/2021). The protocol for the supervision of animals is annexed to this publication.

### 2.2. Urine Assays

Urine was obtained by placing each rat individually in a metabolic cage for 6 h. The urinary creatinine and protein levels were quantified using commercial systems (cat. numbers 1690007 and 1260360, Wiener Lab, Rosario, Argentina) and standardized in a CM250 autoanalyzer (Wiener Lab, Argentina). To determine the TGF-β levels, urine was centrifugated at 3000× *g* for 10 min and then TGF-β was quantified in the supernatants using an ELISA system (cat. number MBS260302, MyBioSource, San Diego, CA, USA) and a Plate Reader Synergy 2 (Agilent Biotek, Santa Clara, CA, USA).

### 2.3. Glomerulus Isolation

The renal cortex was minced and successively strained through 212 μm, 150 μm, 106 μm, and 75 μm sieves. Glomeruli were collected within the finest sieve, achieving a purity of over 90% [30]. The total proteins were obtained by sonication in a lysis buffer (2% SDS, 10% glycerol, 63.5 mM Tris HCl, pH of 6.8) supplemented with the Complete Proteinase Inhibitor, 1 µg/mL pepstatin, and the PhosSTOP^TM^ phosphatase inhibitor (all from Merck KGaA, Darmstadt, Germany). The quantification of protein extracts was achieved using the BCA protein assay kit (Thermo Fisher Scientific, Waltham, MA, USA).

### 2.4. Histological Analysis

A fragment of kidney was fixed in formalin, paraffin-embedded, and sectioned into 5 μm thin slides. For the immunofluorescence assays, the slides were deparaffinized with xylene and hydrated through a battery of solutions with decreasing concentrations of ethanol. The unmasking of antigens was achieved using citrate buffer (10 mM sodium citrate, 0.05% Tween 20, pH of 6.0) by heating the samples in a slow cooker for 30 min, cooling them, and blocking with 2.5% normal horse serum and 1% bovine serum albumin. The samples were incubated with an antibody against A_2B_AR (dilution of 1:250, cat. orb234999, Biorbyt Ltd., Cambridge, UK), anti-α-SMA (dilution of 1:100, cat. sc-130617, Santa Cruz Biotech, Dallas, TX, USA), anti-ZO1 (dilution of 1:100, cat. 61-7300, Thermo Fisher Scientific, USA), anti-nephrin (dilution of 1:100, cat. PA5-106921, Thermo Fisher Scientific, USA), and anti-Snail (dilution of 1:100, cat. ab53519, Abcam, Cambridge, UK) overnight at 4 °C. After washing three times, the samples were incubated with Alexa 488- or Alexa 568-coupled secondary antibodies (dilutions of 1:5000, Thermo Fisher Scientific, USA) for 1 h at room temperature. The samples were visualized in an inverted Zeiss Axio Observer D1 epifluorescence microscope and the captured images were analyzed with the ImageJ v1.54p software.

### 2.5. Purification of Cell Fractions

Pieces (100 mg) of freshly recollected renal cortex from the experimental rat groups were cut into smaller pieces and washed in PBS1× at 500× *g* for 3 min. Cell fractions were obtained using the NE-PER system (cat. 78833, Thermo Fisher Scientific, USA). The buffers were supplemented with the provided Halt Protesase Inhibitor Cocktail (Thermo Fisher Scientific, Waltham, MA, USA). Briefly, tissue pieces were homogenized in 1 mL of prechilled cytoplasmic extraction buffer. The homogenized tissue was transferred to a tissue strainer and centrifugated at 500× *g* for 3 min. The collected supernatant was the cytoplasmic extract. The pellet was resuspended in 650 μL of membrane extraction buffer, incubated for 10 min at 4 °C, and centrifugated at 3000× *g* for 5 min. The pellet was resuspended in 225 μL of nuclear extraction buffer, incubated for 30 min at 4 °C, and centrifugated at 5000× *g* for 5 min. The resulting supernatant was the soluble nuclear extract. The proteins in the nuclear and cytoplasmic fractions were quantified using a BCA protein assay kit (Thermo Fisher Scientific, USA).

### 2.6. Quantification of MCP-1 and CCL3

Glomeruli purified from the experimental groups were incubated in Ham’s F-10 medium for 18 h. The supernatant was collected from a centrifugation step at 800× *g* and filtered through a 0.45 μm mesh. The amounts of MCP-1 and CCL3 were determined using ELISA systems (cat. MBS2701125 and cat. MBS260259 from MyBioSource, San Diego, CA, USA) and a Plate Reader Synergy 2 (Agilent Biotek, Santa Clara, CA, USA). The level of chemokines was normalized to the H3 content in each glomerulus pellet and evaluated by a Western blot.

### 2.7. Cell Culture and Treatments

Immortalized human podocytes generated at the University of Bristol, UK [34], were used at passage 15. The expansion of cells was achieved until the completion of 80% confluence in RPMI medium with 5 mM glucose, 10% fetal bovine serum, an insulin–transferrin–selenium supplement (ITS), and penicillin/streptomycin in proliferating conditions at 33 °C and 5% CO_2_. Then, the cells were seeded at 50,000 cells/cm^2^ on laminin-coated flasks and differentiated at 37 °C and 5% CO_2_ in RPMI medium with 5 mM D-glucose supplemented with 0.5% fetal bovine serum, retinoic acid, vitamin D3, ITS, and penicillin/streptomycin for 14 days, as described previously [26]. For the analysis of the activation of signaling molecules, the podocytes were preincubated with 25 mM D-glucose for 24 h. Then, the cells were exposed to 10 ng/mL of recombinant human TGF-β (cat. 7754-BH, R&D System, Minneapolis, MN, USA) and 50 nM MRS1754 (cat. 2752, Tocris, Minneapolis, MN, USA) in RPMI medium containing 25 mM D-glucose for up to 48 h.

### 2.8. Western Blots

Aliquots containing 50 µg of proteins were fractionated by 10% SDS-PAGE and transferred to a 0.2 μm nitrocellulose membrane. The blots were washed with wash buffer (PBS1×, 0.05% Tween20), blocked for 1 h with 0.5% BSA, and incubated with the primary antibodies anti-ZO-1 (cat. 61-7300, Thermo Fisher Scientific, USA), anti-α-SMA (cat. sc-130617, Santa Cruz Biotech, USA), anti-nephrin (cat. PA5-106921, Thermo Fisher Scientific, USA), anti-Snail (cat. ab53519, Abcam, UK), anti-β-actin (sc-47778, Santa Cruz Biotechnology, USA), anti-phospho-SMAD2 (Ser465/467)/SMAD3 (Ser423/425) (cat. 8828, Cell Signaling, Danvers, MA, USA), anti-phospho-p65-NF-kB (Ser536) (cat. 3033, Cell Signaling, USA), anti-NF-kB p65 (cat. 8242, Cell Signaling, USA), and anti-histone H3 (cat. sc-517578, Santa Cruz Biotech, USA) overnight at 4 °C. The membranes were washed three times and incubated with HRP-coupled secondary antibodies for 1 h at 22 °C. After washing, a chemiluminescence procedure was used for the detection of proteins (Thermo Fisher Scientific, USA). The ratio between the signal of a target protein and the loading controls (β-actin or histone H3) was used to express the relative level.

### 2.9. Chromatin Immunoprecipitation (ChIP)

Chromatin was immunoprecipitated using a modified protocol from [35]. Briefly, trypsinized cells were washed in PBS and fixed with 1% formaldehyde. The cells were then centrifugated and resuspended in lysis buffer (25 mM Hepes with a pH of 7.8, 1.5 mM MgCl_2_, 10 mM KCl, 0.1% NP-40) to release the chromatin. After douncing thoroughly, the chromatin was fragmented into 200–500 bp fragments by sonicating for 150 s using Bioruptor One (Diagenode B01070001) in sonication buffer (50 mM Hepes 7.9, 140 mM NaCl, 1 mM EDTA, 1% Triton X-100, 0.1% sodium deoxycholate, 0.1% SDS). An amount of 25 ug of chromatin was precleared using anti-IgG (cat. 12-370, Milipore, Burlington, MA, USA) and immunoprecipitated with anti-SMAD3 (ca. ab28379, Abcam, UK) and anti-H3K9ac (cat. C15210015, Diagenode, Denville, NJ, USA) overnight at 4 °C. Agarose beads were washed successively with sonication buffer, IP wash buffer (20 mM Tris with a pH of 8.0, 1 mM EDTA, 250 mM LiCl, 0.5% NP-40, 0.5% sodium deoxycholate) and Tris-EDTA buffer. Finally, chromatin was eluted by incubating with elution buffer (50 mM Tris with a pH of 8.0, 1 mM EDTA, 1% SDS) at 65 °C for 15 min, centrifugating, and keeping the supernatant. DNA was purified through the phenol–chloroform method. qPCR was performed by utilizing the Brilliant II SYBR Green qPCR master mix kit (cat. 600828, Agilent, USA). The primers were targeted to comprise SBEs at the promoter regions of fibronectin, collagen type IA2, and the GAPDH genes, using GAPDH as a positive control for acetylated histone immunoprecipitate. An upstream *cis* control region without any SBEs was used as a negative control for SMAD3 immunoprecipitate. The primer sequences were as follows: pFN1, fw–TGAGGACATTGCGTCACCTC, rv–AAAGAAAGGGAGCGGGATGG; Col1a2, fw–CGGAGGTATGCAGACAACGA, rv–TTGGTTCCATTCTTTTTGAGGACG; GAPDH, fw–CGGGATTGTCTGCCCTAAT, rv–GCACGGAAGGTCACGATGT; and control region, fw–GAGCAGCAGCAGGTAAGGAT, rv–AAATGGCTTGGTGCTGTCCT.

### 2.10. Transcriptomic Analysis

The transcriptomic analysis was performed on RNA samples isolated from purified glomeruli from control, DN, and DN + MRS1754 rats using the Illumina platform for RNA library preparation and high-throughput sequencing as previously described [7]. The cellular pathways and processes potentially affected by treatments were identified based on differential transcript contents using the bioinformatic tools Reactome, DAVID v6.8, and the Kyoto Encyclopedia of Genes and Genomes (KEGG) [36,37].

### 2.11. Statistics

The statistical analyses were achieved using the GraphPad Prism 9.0 software. In the Western blot analysis, the data from each animal or cell culture plaque were considered an experimental unit. The determinations in the serum, urines, and glomerulus supernatants were achieved in duplicate. The data were analyzed by an ANOVA and an LSD post hoc test. Statistical differences were stated through *p*-values < 0.05.

## 3. Results

### 3.1. The Antagonism of A_2B_AR Attenuates Renal Alterations in Experimental DN

**Figure 1:**
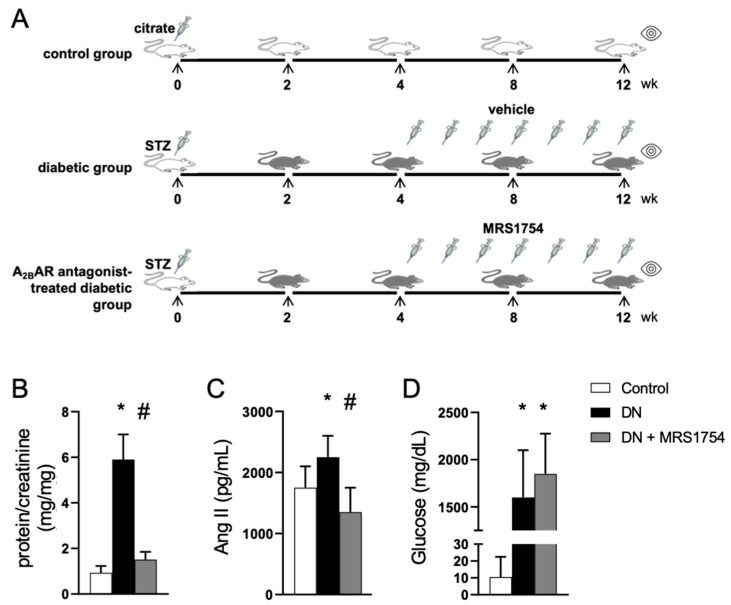
Evaluation of the effect of A_2B_AR antagonism on renal alterations in experimental DN. (**A**) The experimental design consisted of inducing diabetes in rats through the administration of streptozotocin, with control rats receiving citrate buffer. Treatment with the A_2B_AR antagonist MRS1754 (0.5 mg/kg every 48 h) was initiated at week 4 post-diabetes induction. After 8 weeks of treatment, the renal functional and histological alterations were evaluated. The graphs depict the protein-to-creatinine ratio in the urine (**B**), the serum angiotensin 1–8 (Ang II) levels (**C**), and glucosuria (**D**) in the experimental groups. Means ± SD of the samples from 6 animals in each experimental condition are shown. *, *p* < 0.05 vs. control; #, *p* < 0.05 vs. diabetes.

The renal functional alterations and cellular changes associated with diabetes were assessed in STZ-induced diabetic rats, a model of insulin deficiency resulting from pancreatic beta cell depletion. This model mimics the alterations occurring in glomerular cells and serves as a valuable tool for understanding diabetic glomerulopathy [38]. Previously, we determined that early alterations in diabetic kidneys are evident one month after diabetes induction and are concurrent with an increased adenosine level [33]. Therefore, intervening in adenosine signaling at this stage in diabetic rats may allow us to assess its protective effects on the progression of clinical and histopathological signs of DN. In this study, the selective A_2B_AR antagonist MRS1754 was administered at a dose of 0.5 mg/kg to diabetic rats four weeks after diabetes induction, continuing for eight weeks (Figure 1A). We found that experimental diabetes increased the urinary levels of proteins and angiotensin II (Figure 1B,C). The treatment with the A_2B_AR antagonism exerted a protective effect by reducing the development of proteinuria and limiting the activation of the renin–angiotensin system, without affecting the elevated glycemic levels in diabetic animals (Figure 1B–D).

**Figure 2:**
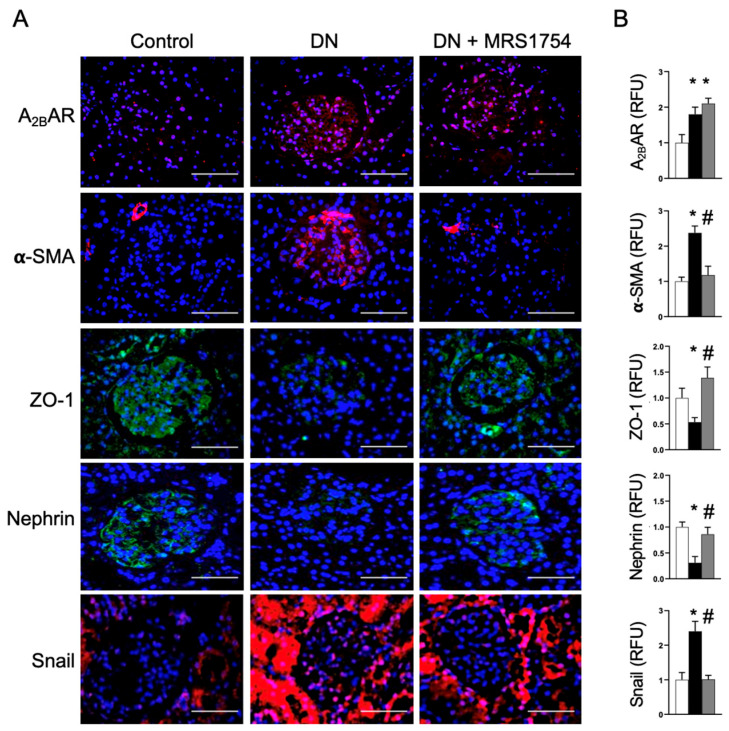
Immunohistochemical analysis of glomerular alterations in diabetic rats. (**A**) The panels show representative immunofluorescent images of the detection of A_2B_AR, α-SMA, ZO-1, nephrin, and Snail in renal tissue from control rats, diabetic nephropathy (DN) rats, and diabetic rats treated with the A_2B_AR antagonist MRS1754 (DN + MRS1754). The images emphasize changes in the levels of these proteins within the glomerulus. Nuclear staining with DAPI is in blue. The bar indicates 50 µm. (**B**) The graphs correspond to the means ± SD of relative fluorescent units (RFUs) of a quantitative analysis of A_2B_AR, α-SMA, ZO-1, nephrin, and Snail in glomeruli from the control (white bar), diabetic (black bar), and MRS1754-treated diabetic (grey bar) rat groups. The means in the control group were normalized to 1. Images of 12 glomeruli from 2 control, 4 diabetic, and 4 treated diabetic rats were analyzed. *, *p* < 0.05 vs. control; #, *p* < 0.05 vs. diabetes.

An immunohistochemical analysis, used to evaluate renal repercussions, showed the induction of A_2B_AR in the diabetic glomerulus (Figure 2), which was not affected by the MRS1754 treatment. Importantly, the induction of the mesenchymal-like transformation marker α-SMA was evident in the glomeruli of diabetic rats. However, treatment with the A_2B_AR antagonist markedly prevented an increase in α-SMA (Figure 2), indicating that the treatment interferes with the profibrogenic activation of cells.

### 3.2. The Antagonism of A_2B_AR Attenuates Podocyte Dedifferentiation

The dedifferentiation of podocytes occurs in DN, and this phenotypic change is associated with the progressive disruption of the filtration barrier and glomerulosclerosis. An immunohistochemical analysis of control rat kidneys showed the glomerular expression of nephrin and ZO-1, markers of differentiated visceral epithelial podocytes. However, their levels were reduced after twelve weeks of diabetes induction (Figure 2). These changes were prevented in diabetic rats treated with MRS1754 (Figure 2). Previous studies have linked the activation of the Snail transcription factor (encoded by the *snai1* gene) with renal fibrosis, observed in Snail-inducible transgenic mouse and UUO mouse kidneys [6]. Snail potently induces the mesenchymal-like transition in epithelial cells, thus linking this process to renal fibrogenesis [6]. In diabetic rats, Snail induction was evident in epithelial tubule cells and within glomeruli (Figure 2). This induction was attenuated in the epithelial tubule cells and prevented within the glomeruli of rats treated with the A_2B_AR antagonist (Figure 2).

**Figure 3:**
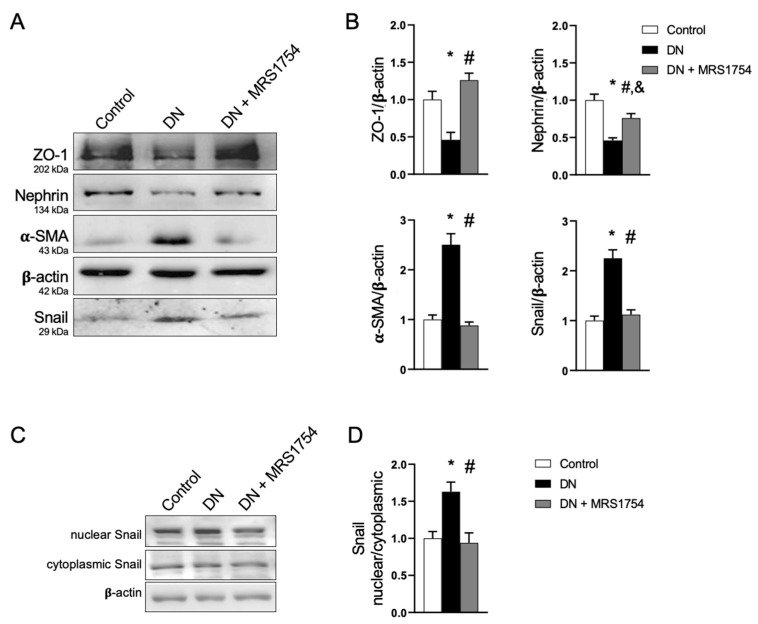
A_2B_AR antagonism prevents the loss of podocyte epithelial markers in diabetic glomerulopathy. (**A**) Representative Western blot analysis showing the levels of epithelial ZO-1 and nephrin, as well as mesenchymal α-SMA and Snail, in purified glomeruli from the different rat groups. (**B**) The graphs represent the means and SD of the levels of epithelial and mesenchymal markers relative to β-actin. The means in the control group were normalized to 1. (**C**) Representative Western blot analysis of the Snail distribution in cell fractions purified from the kidneys of experimental rat groups. (**D**) The graph represents the ratio of the Snail subcellular distribution. The mean in the control was normalized to 1. The number of animals analyzed in each experimental group was 5 in (**B**) and 2 in (**D**). *, *p* < 0.05 vs. control; #, *p* < 0.05 vs. DN; &, *p* < 0.05 vs. control.

A Western blot analysis of purified glomeruli from rat kidneys confirmed the preservation of the epithelial markers nephrin and ZO-1 in A_2B_AR antagonist-treated diabetic rats, along with the attenuation of the mesenchymal markers (Figure 3A). MRS1754 also prevented the induction of Snail in the glomeruli of diabetic rats (Figure 3A). Additionally, an analysis of the cell fractions obtained from the renal tissue of diabetic rats showed that A_2B_AR antagonism decreases the nuclear content of Snail, indicating a reduction in its activity (Figure 3B).

**Figure 4:**
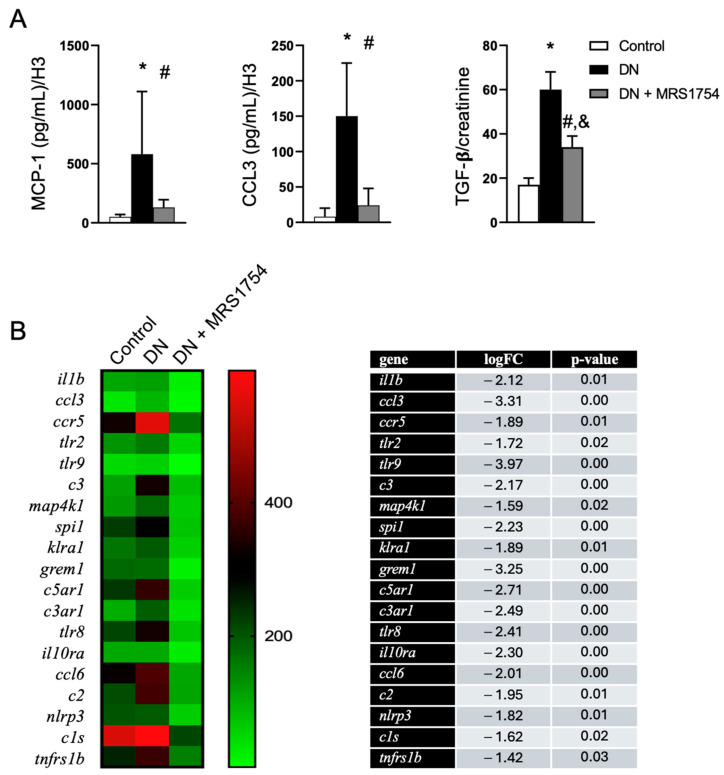
A_2B_AR antagonism attenuates inflammatory mediators in diabetic rats. (**A**) The graphs depict the levels of the chemokines MCP-1 and CCL3 released from glomeruli and the urinary TGF-β levels in the rat experimental groups. *n* = 6 samples per duplicate. *, *p* < 0.05 vs. control; #, *p* < 0.05 vs. DN; &, *p* < 0.05 vs. control. (**B**) The heat map highlights changes in the transcript levels of selected inflammation-related genes in the experimental rat groups. The table shows values related to a decrease in the transcripts in the DN + MRS1754 group vs. the DN group.

Renal cell dedifferentiation during fibrogenesis has been linked to a secretory phenotype that produces proinflammatory cytokines and fibrotic factors, which orchestrate immune cell infiltration and myofibroblast generation [6,7]. We evaluated the production of some of these factors that may be affected in the glomeruli. The secretion of MCP-1 and CCL3 from glomeruli, as well as urinary TGF-β levels, were increased in diabetic rats; however, A_2B_AR antagonism blocked their elevation (Figure 4A). A further analysis of the transcriptional profiles in glomeruli purified from the rat groups revealed that the MRS1754 treatment strongly affected the expression of several inflammation-related genes associated with the progression of DN, including the IL1β and NLRP3 components of the inflammasome, chemokines, complement factors and receptors, and gremlin (Figure 4B). A list of downregulated transcripts belonging to the immune system processes in the glomeruli of diabetic rats treated with MRS1754 is shown in Table S1. In conclusion, we suggest that A_2B_AR antagonism protects against the cellular dedifferentiation that occurs during the progression of diabetic glomerulopathy.

### 3.3. The Antagonism of A_2B_AR Blocks TGF-β Signaling in Podocytes

**Figure 5:**
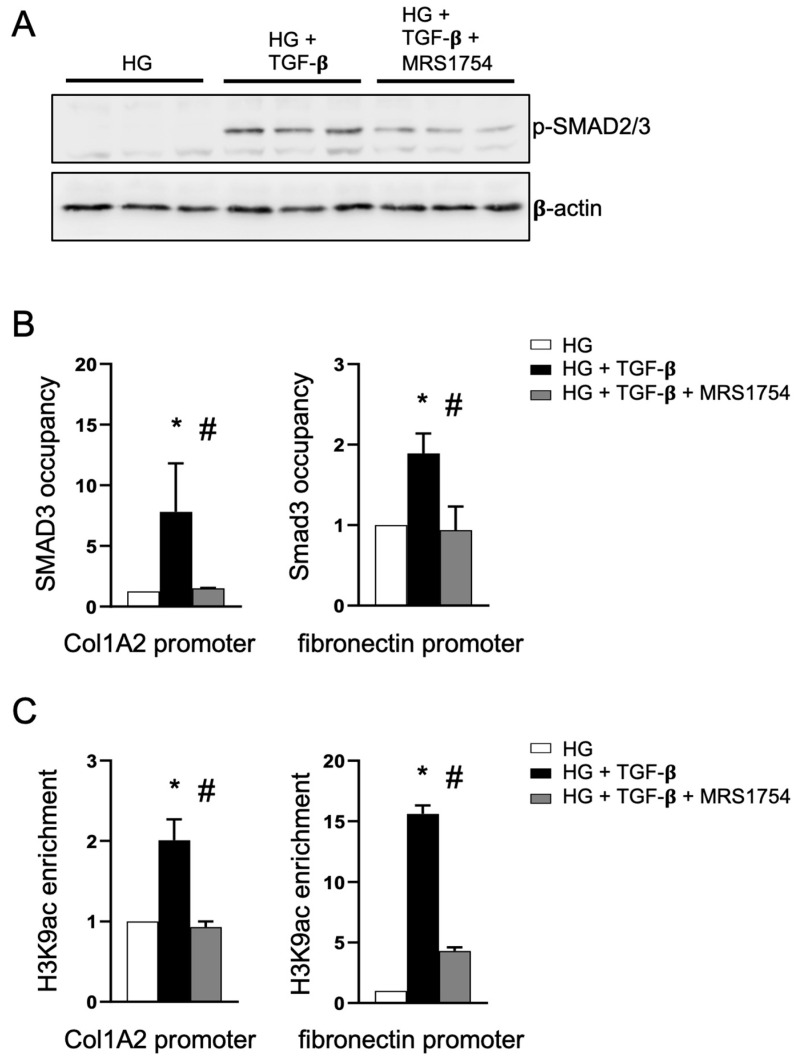
The antagonism of A_2B_AR blocks TGF-β/SMAD3 signaling in podocytes. Differentiated podocytes were incubated in medium containing 25 mM D-glucose (HG) and treated with 10 ng/mL TGF-β and 50 nM MRS1754. (**A**) Upper panel shows the detection of activated phospho-SMAD2/-3 following 0.5 h of treatments in total protein extracts from three independent experiments through Western blot. Lower panel is detection of β-actin as loading control; *n* = 3. (**B**) Podocytes were treated as above for 48 h, refreshing TGF-β and MRS1754 at 24 h, and harvested for a ChIP-qPCR analysis. The graphs depict normalized means and the SEM occupancy of SMAD3 to the collagen type IA2 and fibronectin promoters; *n* = 5. *, *p* < 0.05 vs. HG; #, *p* < 0.05 vs. TGF-β. (**C**) The graphs show normalized means and the SEM enrichment of H3K9ac at SMAD3 target promoters; *n* = 5. *, *p* < 0.05 vs. HG; #, *p* < 0.05 vs. TGF-β.

Since renal fibrosis and the mesenchymal-like phenotypic transformation of renal cells in DN are linked to the pathogenic cascade of TGF-β, we evaluated the consequences of A_2B_AR antagonism on these cytokine signaling properties. We used immortalized human podocytes as the cellular model [34], as this cell type expresses A_2B_AR [26] and exposure to high D-glucose levels leads to increased extracellular adenosine [25]. The treatment of podocytes with TGF-β resulted in an increase in phosphorylated SMAD2/-3, indicating the activation of the canonical pathway (Figure 5A). Notably, A_2B_AR antagonism attenuated the TGF-β-mediated activation of SMAD2/-3 in podocytes (Figure 5A). Since activated SMADs function as transcription factors, we assessed whether A_2B_AR antagonism affects SMAD3 transactivation activity by examining its binding to target sites on collagen type 1A and fibronectin gene promoters. Chromatin immunoprecipitation (ChIP) assays revealed an increase in SMAD3 binding to these TGF-β target gene promoters, which was strongly inhibited by A_2B_AR antagonism (Figure 5B). Furthermore, the TGF-β-induced increase in histone H3K9 acetylation was also reduced by MRS1754, indicating that A_2B_AR antagonism impacts SMAD3 transactivation activity (Figure 5B).

**Figure 6:**
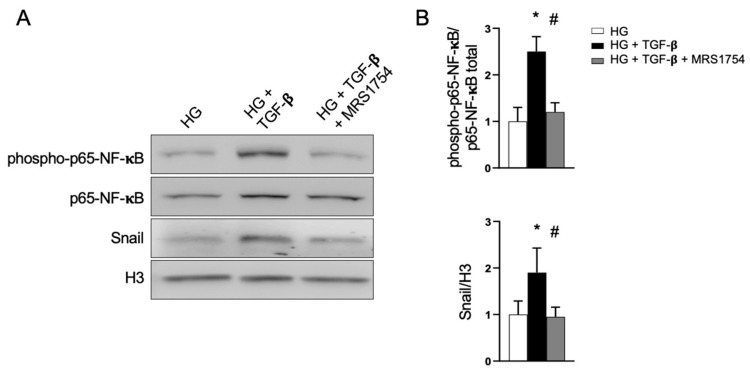
Non-canonical p65-NF-κB activation by TGF-β is blocked by MRS1754 in podocytes. Differentiated podocytes were incubated in medium containing 25 mM D-glucose (HG) and treated with TGF-β (10 ng/mL) and MRS1754 (50 nM) for 24 h. (**A**) The levels of phosphorylated active p65-NF-κB, total p65-NF-κB, Snail, and histone H3 were evaluated in the total protein extracts from the podocytes through Western blots. (**B**) The graphs depict the means of the ratio between phosphorylated and total p65-NF-κB levels and the ratio between Snail relative to histone H3 in treated podocytes; *n* = 3. *, *p* < 0.05 vs. HG; #, *p* < 0.05 vs. TGF-β.

Because several inflammatory factors produced in the glomerulus and affected by MRS1754 treatment in diabetic rats are regulated by p65-NF-κB activation (Figure 4A), we investigated the effects of A_2B_AR antagonism on the non-canonical activation of this signaling molecule. In vitro assays in podocytes demonstrated that the levels of the active Ser536-phosphorylated p65-NF-κB increase upon TGF-β exposure. However, cotreatment with the A_2B_AR antagonist prevented its activation (Figure 6). Consistent with this inhibitory effect, the TGF-β-induced increase in Snail expression in podocytes was also prevented by MRS1754 (Figure 6). Thus, we conclude that A_2B_AR antagonism directly interferes with cellular signaling downstream of TGF-β in podocytes.

## 4. Discussion

Altered adenosine signaling properties due to increased adenosine levels have been linked to key features of diabetic glomerulopathy, such as proteinuria and fibrogenesis. Indeed, the use of adenosine deaminase, which decreases adenosine by converting it to inosine, has been shown to prevent α-SMA induction in experimental diabetic nephropathy [30]. Furthermore, an increase in adenosine levels appears to occur concurrently with the induction of the low-affinity A_2B_AR in the glomerulus, as observed in the diabetic kidneys of rats and in patients affected by DN [4]. This emphasizes the need to analyze the consequences of an A_2B_ receptor blockade. This study demonstrates that the beneficial effects of A_2B_AR antagonism may occur through the mitigation of phenotypic transformation in podocytes, which relates to the emergence of proteinuria and the production of inflammatory mediators that drive diabetic glomerulopathy. Mechanistically, these effects may result from interference with the TGF-β signaling pathways involving SMAD3 and NF-κB, directly within podocytes. The recent KDIGO guidelines recommend the use of additional drugs with potential renoprotective effects, such as SGLT2 inhibitors. This highlights the urgent need for new therapeutic options for patients with DN, as the progression of this renal disease imposes significant economic burdens on healthcare systems, mainly due to organ replacement therapies and indirect costs related to morbidity, a shortened productive lifespan, and early mortality [39,40]. Translating A_2B_AR antagonism into the clinical setting could represent a promising avenue for DN treatment. Some advantages of targeting A_2B_AR include its induction during pathology and the feasibility of pharmacological targeting using hydrophilic drugs, given its distribution on the cell surface. Of interest, a novel A_2B_AR antagonist is currently advancing through clinical trials for non-small cell lung cancer, demonstrating good tolerability and no dose-limiting toxicities [41], which opens the possibility of evaluating its efficacy in other pathologies.

Considering TGF-β as a master pathogenic factor in DN, an important advantage of A_2B_AR antagonism lies in its ability to intervene in key signaling molecules downstream of TGF-β in podocytes, which are strongly linked to diabetic glomerulopathy. Previous studies that directly targeted the TGF-β ligand with neutralizing antibodies have yielded disappointing results for DN treatments, primarily due to the pleiotropic effects of this cytokine on human physiology [42]. Therefore, it has been concluded that therapeutic interventions should aim to selectively modulate TGF-β actions involved in DN. The TGF-β/SMAD3 pathway has garnered particular interest in the progression of diabetic glomerulopathy because it is mainly associated with podocyte apoptosis and loss [43,44]. Indeed, endogenous signaling regulators, such as gremlin and LRG1, can influence TGF-β effects in diabetic glomerulopathy by enhancing SMAD3 signaling in podocytes [24,45]. Surprisingly, selectively interfering with SMAD3 signaling can attenuate glomerulosclerosis; however, this has shown minimal effects in preventing proteinuria in experimental DN [43,46]. This highlights the need for further research into signaling networks to develop targeted therapies against pathogenic TGF-β. Notably, A_2B_AR antagonism prevents the phenotypic dedifferentiation of podocytes and exerts a beneficial antiproteinuric effect in diabetic rats. Since the inhibition of SMAD3 activity alone appears weakly related to protection against proteinuria, other complementary mechanisms may be involved in mediating the beneficial effects of the A_2B_AR blockade. Podocyte function critically depends on maintaining cytoskeletal plasticity to support foot process integrity and on preserving proteins essential for the slit diaphragm structure. Our findings suggest that A_2B_AR antagonism prevents the decline of nephrin in the glomerular filtration barrier of diabetic rats, which could be a key factor in its antiproteinuric effects. Additionally, the pharmacological blockade of A_2B_AR has been shown to affect focal adhesion kinase activation in experimental DN and alter the podocyte adhesion dynamics in vitro. This may contribute to preventing foot process effacement and proteinuria [26]. Moreover, A_2B_AR antagonism may interfere with the TGF-β-mediated, non-canonical activation of p65-NF-κB, which could further ameliorate diabetic glomerulopathy. Although the link between NF-κB activation and proteinuria in DN remains unclear, studies have demonstrated that NF-κB is activated in multiple renal cell types, including podocytes, in human proteinuric diseases of a different etiology, such as lupus nephritis, minimal change disease, and idiopathic membranous nephropathy [47]. Furthermore, podocyte-specific transgenic mice that continuously inhibit NF-κB signaling show reduced proteinuria in adriamycin-induced nephropathy [48]. Therefore, the inhibitory effect of A_2B_AR antagonism on NF-κB activation may also contribute to its protective role against proteinuria in diabetic rats.

On the other hand, abundant evidence indicates that overactive NF-κB plays a key role in renal fibrogenesis [49]. NF-κB has been linked to the activation and recruitment of immune cells and is associated with the induction of a range of inflammatory mediators, as well as crosstalk with increased TGF-β production [22,49]. Furthermore, NF-κB activation appears necessary for TGF-β-triggered cellular responses, such as a mesenchymal-like transition in certain cell types [50]. Therefore, the phenotype observed in diabetic animals, characterized by the prevention of the mesenchymal-like transition, a reduction in inflammatory mediators, and decreased TGF-β levels, aligns with the blockade of NF-κB activation achieved through A_2B_AR antagonism. Previous studies have shown that A_2B_AR antagonism inhibits monocyte and macrophage infiltration into the glomerulus and prevents their transformation into myofibroblasts [7,33], supporting the notion that the cellular transition toward an inflammatory and profibrotic program was effectively disrupted. Importantly, the TGF-β-mediated induction of Snail in podocytes in vitro was also inhibited by MRS1754, highlighting the protective effect of A_2B_AR antagonism on cell dedifferentiation directly within this cell type. A recent study described that the inhibition of A_2B_AR decreased NF-κB in human renal tubular epithelial cells, which was protective against nephropathy in db/db mice [51]. Thus, A_2B_AR antagonism may be a tool to target cell types susceptible to phenotypic changes that orchestrate renal fibrosis.

Among the beneficial effects resulting from the A_2B_AR blockade, at the glomerular levels, we emphasize the downregulation of complement factors and receptors. Increasing evidence links complement system activation to DN [52], with this activation being strongly associated with chronic unresolved inflammation, cell senescence, and renal fibrosis [53]. This area holds clinical significance, as the current treatments for DN, such as RAS blockers, do not target complement activation or the associated senescence pathways [54]. Another notable benefit of A_2B_AR antagonism is the significant attenuation of gremlin, a factor the potentiates TGF-β actions; it is elevated in fibrotic areas of diabetic kidneys and increases in podocytes cultured in elevated glucose levels [45]. Gremlin has been implicated in podocyte injury by enhancing deleterious TGF-β effects and decreasing nephrin and synaptopodin expression [45]. Additionally, gremlin may exert pathogenic effects by acting as an agonist of VEGF receptor 2, leading to the activation of the NF-κB pathway and the upregulation of proinflammatory factors, as well as the infiltration of immune cells [55]. Moreover, gremlin can antagonize bone morphogenetic proteins (BMPs), such as BMP7, which are negative regulators of TGF-β signaling [56]. Recent studies have elucidated that the autocrine and paracrine actions of TGF-β regulators are crucial in the progression of diabetic glomerulopathy [21,24,57]. Therefore, restoring this balance through A_2B_AR antagonism may yield benefits beyond the mere alleviation of podocyte injury, potentially impacting broader pathogenic pathways involved in diabetic nephropathy.

## 5. Conclusions

We conclude that antagonizing A_2B_AR may be an effective strategy to alleviate diabetic glomerulopathy by directly modulating TGF-β pathogenic signaling in podocytes and potentially improving glomerular function through the attenuation of phenotypic dedifferentiation and the associated inflammatory cascade in DN.

## Data Availability

The raw data supporting the conclusions of this article will be made available by the authors on request.

## Supplementary Materials

The following supporting information can be downloaded at: https://www.mdpi.com/article/10.3390/cells14120890/s1, Table S1: List of downregulated transcripts related to the immune system in glomeruli of diabetic nephropathy rats treated with MRS1754.

## Abbreviations

The following abbreviations are used in this manuscript:

A_2B_AR	Adenosine A_2B_ receptor
DN	Diabetic nephropathy
RAS	Renin–angiotensin system
α-SMA	Alfa-smooth muscle actin
STZ	Streptozotocin
TGF-β	Transforming growth factor beta 1
NF-κB	Nuclear factor kappa B
SMAD	Small mother against decapentaplegic
ZO-1	Zonula occludens 1
IL-1β	Interleukin 1 beta
MCP-1	Monocyte chemoattractant protein 1
MAPK	Mitogen-activated protein kinase
KDIGO	Kidney Disease: Improving Global Outcomes
