# Supplementary material for: Adenosine A2B Receptor Antagonism Interferes with TGF-β Cellular Signaling Through SMAD2/-3 and p65-Nf-κB in Podocytes and Protects from Phenotypical Transformation in Experimental Diabetic Glomerulopathy
Source: Cells. 2025 Jun 12;14(12):890. doi: 10.3390/cells14120890 (PMC12190782; doi:10.3390/cells14120890)
Supplement: Supplementary file 1 [file cells-14-00890-s001.zip › cells-3652008-supplementary.pdf]

Table S1. List of downregulated transcripts related to the immune system in glomeruli of diabetic nephropathy rats treated with MRS1754.

Gene Symbol	Transcript	logFC	logCPM	p-value
Clec4d	NM_001003707	-7,90	0,78	0,00
S100g	NM_012521	-7,55	0,48	0,00
Cd163	NM_001107887	-7,09	0,09	0,00
Rrh	NM_001107726	-6,90	-0,06	0,00
Cd300lb	NM_001167664	-6,90	-0,06	0,00
Slamf6	NM_001191932	-6,68	-0,24	0,00
Igfbp6	NM_013104	-6,68	-0,24	0,00
Slc22a7	NM_053537	-6,68	-0,24	0,00
Slc25a47	NM_001001509	-6,42	-0,43	0,01
Vtcn1	NM_001024244	-6,42	-0,43	0,01
Nkr-p1c	NM_001040189	-6,42	-0,43	0,01
Pon1	NM_032077	-6,42	-0,43	0,01
Fga	NM_052797	-6,42	-0,43	0,01
Park7	NM_057143	-6,42	-0,43	0,01
Ccl21	NM_001008513	-6,10	-0,66	0,02
Lgals12	NM_001106333	-6,10	-0,66	0,02
Lypd2	NM_001130545	-6,10	-0,66	0,02
Kcnn4	NM_001270701	-6,10	-0,66	0,02
Hdc	NM_017016	-6,10	-0,66	0,02
Arg1	NM_017134	-6,10	-0,66	0,02
Gper1	NM_133573	-6,10	-0,66	0,02
Acvr1c	NM_139090	-6,10	-0,66	0,02
Kir3dl1	NM_181479	-6,10	-0,66	0,02
Tlr9	NM_198131	-3,97	1,83	0,00
Trem2	NM_001106884	-3,72	2,15	0,00
Clec4e	NM_001005897	-3,53	0,58	0,00
Mbl1	NM_012599	-3,51	1,43	0,00
Ubash3a	NM_001107620	-3,40	0,47	0,00
Slc5a10	NM_001107007	-3,35	3,98	0,00
Ccl3	NM_013025	-3,31	2,49	0,00
Grem1	NM_019282	-3,25	3,26	0,00
Rbp4	NM_013162	-3,24	3,08	0,00
Batf	NM_001106748	-3,08	0,23	0,01
Egr3	NM_017086	-3,08	0,23	0,01
Cxcl9	NM_145672	-3,08	0,23	0,01
Fabp4	NM_053365	-2,97	4,87	0,00
Marchf1	NM_001135838	-2,94	1,48	0,00
Sectm1a	NM_001013043	-2,93	0,94	0,00
Vtn	NM_019156	-2,89	0,09	0,02
Serpina1	NM_022519	-2,89	0,09	0,02
Cyp26b1	NM_181087	-2,79	8,04	0,00
Egr2	NM_053633	-2,72	1,69	0,00
C5ar1	NM_053619	-2,71	4,55	0,00
Wnt7b	NM_001009695	-2,70	0,77	0,01
Gpx2	NM_183403	-2,70	2,43	0,00
Adgre1	NM_001007557	-2,68	4,82	0,00
Arhgap9	NM_001012198	-2,67	-0,07	0,04
Trim29	NM_001106815	-2,67	-0,07	0,04
Il12rb1	NM_001170604	-2,67	-0,07	0,04

Mpeg1	NM_001305460	-2,67	-0,07	0,04
Ebi3	NM_001109421	-2,63	1,61	0,00
RT1-Bb	NM_001004084	-2,61	7,28	0,00
RT1-M2	NM_001001717	-2,60	2,70	0,00
Ncf4	NM_001127304	-2,59	3,75	0,00
Mep1b	NM_013183	-2,59	3,75	0,00
Bcat1	NM_017253	-2,58	2,96	0,00
Mlc1	NM_001108105	-2,54	2,49	0,00
Lyz2	NM_012771	-2,53	8,96	0,00
RGD1559482	NM_001168285	-2,52	3,36	0,00
C3ar1	NM_032060	-2,49	3,67	0,00
Gpnmb	NM_133298	-2,48	5,62	0,00
Tspan1	NM_001004236	-2,47	1,78	0,00
Ptprc	NM_001109890	-2,47	1,78	0,00
Gpr132	NM_001170595	-2,47	1,78	0,00
Siglec5	NM_001106249	-2,45	3,09	0,00
Fcmr	NM_001014843	-2,44	3,28	0,00
Cd4	NM_012705	-2,43	5,74	0,00
Vav1	NM_012759	-2,42	3,82	0,00
Lpcat2	NM_001271344	-2,42	3,60	0,00
Tlr8	NM_001101009	-2,41	4,47	0,00
Cd68	NM_001031638	-2,38	5,15	0,00
Cd84	NM_001192006	-2,37	3,41	0,00
Angptl4	NM_199115	-2,35	2,64	0,00
Adap1	NM_133567	-2,35	3,11	0,00
Vipr1	NM_012685	-2,33	3,60	0,00
Itgad	NM_031691	-2,30	3,35	0,00
Il10ra	NM_057193	-2,30	2,85	0,00
S1pr5	NM_021775	-2,30	2,10	0,00
Hcst	NM_001005900	-2,29	0,47	0,04
Mall	NM_001014182	-2,29	0,47	0,04
RT1-DOa	NM_183051	-2,29	0,47	0,04
Rgs1	NM_019336	-2,29	2,96	0,00
Lilrb4	NM_001013894	-2,28	4,47	0,00
Lilrb3	NM_031713	-2,27	3,81	0,00
Fcgr2a	NM_053843	-2,27	3,15	0,00
Procr	NM_001025733	-2,27	2,43	0,00
Fgr	NM_024145	-2,26	4,97	0,00
Pld4	NM_001126288	-2,25	5,61	0,00
Prkcb	NM_012713	-2,23	2,92	0,00
Cd72	NM_001015016	-2,23	2,40	0,00
Spi1	NM_001005892	-2,23	4,36	0,00
Cd37	NM_017124	-2,22	3,59	0,00
Cx3cr1	NM_133534	-2,22	3,94	0,00
Lst1	NM_022634	-2,22	2,60	0,00
Tbx21	NM_001107043	-2,21	1,58	0,01
Ncr3	NM_181822	-2,21	0,41	0,05
Fcgr1a	NM_001100836	-2,20	5,12	0,00
Fcgr3a	NM_207603	-2,18	4,44	0,00
Clec12a	NM_001134716	-2,18	3,91	0,00
C1qa	NM_001008515	-2,18	6,35	0,00
C3	NM_016994	-2,17	4,50	0,00
LOC685157	NM_001115044	-2,17	5,27	0,00

Fcer1g	NM_001131001	-2,16	5,95	0,00
Mrc1	NM_001106123	-2,16	5,59	0,00
Pla2g2d	NM_001013428	-2,16	4,65	0,00
Ctss	NM_017320	-2,16	6,54	0,00
Tyrobp	NM_212525	-2,16	5,97	0,00
Lcp1	NM_001012044	-2,13	7,51	0,00
Cd300le	NM_001202463	-2,13	3,70	0,00
Arl11	NM_001013433	-2,12	3,36	0,00
Il1b	NM_031512	-2,12	2,45	0,01
Aif1	NM_017196	-2,10	5,57	0,00
Matk	NM_021859	-2,10	2,81	0,00
Nckap1l	NM_001108119	-2,10	5,47	0,00
Hcls1	NM_001011898	-2,09	5,13	0,00
Cybb	NM_023965	-2,07	5,86	0,00
Adcy7	NM_053396	-2,07	3,25	0,00
Ptx3	NM_001109536	-2,07	1,47	0,01
Tagap	NM_001309449	-2,07	2,78	0,01
Ccr1	NM_020542	-2,07	4,41	0,00
Fyb	NM_001109176	-2,05	4,87	0,00
Lilrc2	NM_001100123	-2,05	1,16	0,02
Trem1	NM_001106885	-2,05	1,16	0,02
C1qb	NM_019262	-2,03	6,34	0,00
Lsp1	NM_001025420	-2,02	5,61	0,00
Plek	NM_001025750	-2,02	4,19	0,00
Itgal	NM_001033998	-2,02	5,73	0,00
Ccl6	NM_001004202	-2,01	4,76	0,00
Clec10a	NM_022393	-2,01	5,05	0,00
Slamf8	NM_001105973	-2,01	2,74	0,01
Myo1f	NM_001108076	-2,01	5,29	0,00
Fgd2	NM_001107617	-2,00	4,01	0,00
Blnk	NM_001025767	-2,00	3,47	0,01
Fcnb	NM_053634	-2,00	3,59	0,01
Lat2	NM_173840	-1,99	1,42	0,02
Csf1r	NM_001029901	-1,97	7,25	0,00
Spn	NM_001271086	-1,97	6,16	0,00
Mefv	NM_031634	-1,96	2,81	0,01
Itgam	NM_012711	-1,95	3,78	0,01
C2	NM_172222	-1,95	4,74	0,01
Myo1g	NM_001134843	-1,95	4,69	0,01
Lcp2	NM_130421	-1,94	5,20	0,01
Clec4a2	NM_001005880	-1,91	1,36	0,02
Ch25h	NM_001025415	-1,90	4,25	0,01
Ccr5	NM_053960	-1,89	5,30	0,01
Bank1	NM_001047918	-1,88	1,58	0,02
Itgb2	NM_001037780	-1,88	6,37	0,01
Siglec1	NM_001107777	-1,88	3,87	0,01
C1qc	NM_001008524	-1,87	6,74	0,01
Clec4a3	NM_001005891	-1,87	4,96	0,01
Klra1	NM_001009486	-1,86	3,81	0,01
Sash3	NM_001134992	-1,86	3,70	0,01
Ptpn7	NM_145683	-1,86	2,91	0,01
Tlr1	NM_001172120	-1,85	3,94	0,01
RT1-Db1	NM_001008884	-1,84	8,41	0,01

Ptpn6	NM_053908	-1,83	5,56	0,01
Evi2b	NM_001271482	-1,83	3,83	0,01
Lilrb3l	NM_001037357	-1,83	5,92	0,01
Cfd	NM_001077642	-1,83	2,24	0,02
Cd180	NM_001106405	-1,83	3,92	0,01
Pid1	NM_001110493	-1,82	1,29	0,03
Dok3	NM_001107336	-1,82	2,97	0,01
Ctsz	NM_183330	-1,82	5,85	0,01
Nlrp3	NM_001191642	-1,82	3,82	0,01
Cst7	NM_001106523	-1,81	2,69	0,01
Lilra5	NM_001076793	-1,80	3,76	0,01
Coro1a	NM_130411	-1,79	6,18	0,01
Pde1a	NM_030871	-1,79	3,75	0,01
Sh3bp2	NM_001100684	-1,79	3,88	0,01
Nod2	NM_001106172	-1,78	2,20	0,02
Cd300a	NM_001205348	-1,78	4,00	0,01
RT1-DOb	NM_001008846	-1,77	2,66	0,02
Cd36	NM_031561	-1,77	7,22	0,01
Cd74	NM_013069	-1,77	10,01	0,01
Hck	NM_013185	-1,77	4,68	0,01
Mmp12	NM_053963	-1,75	4,53	0,01
Tlr7	NM_001097582	-1,74	4,42	0,01
Slamf9	NM_001105971	-1,74	3,27	0,02
Slco4a1	NM_133608	-1,74	3,21	0,02
Folr2	NM_001106283	-1,74	2,64	0,02
Vpreb1	NM_001108845	-1,74	2,53	0,02
Laptm5	NM_053538	-1,74	6,15	0,01
Csad	NM_001134454	-1,73	1,47	0,04
Tmem255a	NM_182822	-1,73	1,47	0,04
Ccdc85a	NM_001191553	-1,73	1,23	0,04
Rbm24	NM_001191100	-1,73	3,13	0,02
Nfe2	NM_001012224	-1,73	0,94	0,05
Dlg2	NM_022282	-1,73	0,94	0,05
Tlr2	NM_198769	-1,72	3,55	0,02
Csf3r	NM_001106685	-1,72	3,44	0,02
Gclc	NM_012815	-1,72	8,68	0,01
Slc7a13	NM_001012100	-1,71	6,11	0,01
RT1-Ba	NM_001008831	-1,70	8,38	0,01
Fcna	NM_031348	-1,69	2,27	0,03
RT1-Da	NM_001008847	-1,69	8,93	0,01
Lyl1	NM_001007677	-1,69	3,03	0,02
Lpxn	NM_001009649	-1,68	3,41	0,02
Pik3cd	NM_001108978	-1,67	4,97	0,01
Gnmt	NM_017084	-1,67	3,45	0,02
Serpinb1a	NM_001031642	-1,66	4,32	0,02
Csf2rb	NM_133555	-1,66	6,56	0,01
Cd83	NM_001108410	-1,65	4,23	0,02
Baiap2l2	NM_001271220	-1,65	4,32	0,02
Cth	NM_017074	-1,65	5,94	0,02
Cfh	NM_130409	-1,64	5,76	0,02
Tnfaip8l2	NM_001014039	-1,64	4,42	0,02
Esm1	NM_022604	-1,64	5,99	0,02
Lgals3	NM_031832	-1,64	5,17	0,02

Trpm2	NM_001011559	-1,63	4,08	0,02
Rac2	NM_001008384	-1,63	5,37	0,02
Cfp	NM_001106757	-1,63	5,46	0,02
Selplg	NM_001013230	-1,62	3,28	0,02
C1s	NM_138900	-1,62	5,46	0,02
Cyp4b1	NM_016999	-1,62	4,59	0,02
Incenp	NM_001106335	-1,61	4,94	0,02
Itga4	NM_001107737	-1,61	5,19	0,02
Card9	NM_022303	-1,61	2,97	0,03
Il1rn	NM_022194	-1,60	4,02	0,02
Prkcb	NM_001172305	-1,60	2,20	0,04
Mt2A	NM_001137564	-1,60	7,35	0,02
Glipr1	NM_001011987	-1,60	4,83	0,02
Gas7	NM_053484	-1,59	4,44	0,02
Kcnj10	NM_031602	-1,59	1,58	0,05
Map4k1	NM_001106243	-1,59	3,74	0,02
Steap4	NM_001044265	-1,58	3,15	0,03
Igsf6	NM_133542	-1,58	4,10	0,02
Defb1	NM_031810	-1,57	4,65	0,02
Rasd1	NM_001270954	-1,57	2,61	0,03
Slc11a1	NM_001031658	-1,56	5,20	0,02
Cd53	NM_012523	-1,56	5,65	0,02
Sectm1b	NM_199082	-1,56	2,50	0,04
Gpr65	NM_001106751	-1,55	2,17	0,05
Fam131b	NM_001025046	-1,54	2,39	0,04
Rgs10	NM_019337	-1,54	4,40	0,03
Fmnl1	NM_001105846	-1,53	5,76	0,02
Ada	NM_130399	-1,52	7,59	0,02
Btk	NM_001007798	-1,52	2,47	0,04
Grem2	NM_001105974	-1,52	2,47	0,04
Cd244	NM_022259	-1,50	3,91	0,03
Sele	NM_138879	-1,50	3,44	0,04
Scin	NM_198748	-1,50	3,49	0,04
Pstpip2	NM_001271281	-1,50	2,36	0,05
Itgb7	NM_013171	-1,50	2,56	0,04
Ly86	NM_001106128	-1,49	3,66	0,03
Capg	NM_001013086	-1,48	4,65	0,03
Ikzf1	NM_001107237	-1,48	3,52	0,04
Cdo1	NM_052809	-1,48	3,32	0,04
Gpr183	NM_001109386	-1,48	2,95	0,04
P2ry6	NM_057124	-1,48	2,95	0,04
Sfrp1	NM_001276712	-1,48	3,67	0,04
Unc93b1	NM_001108513	-1,44	5,86	0,03
Clec4a1	NM_001005890	-1,43	5,41	0,03
Prkcq	NM_001276721	-1,43	3,53	0,04
Clec7a	NM_001173386	-1,43	4,99	0,04
Tnfrsf1b	NM_130426	-1,42	4,82	0,04
Arhgap22	NM_001107297	-1,41	3,54	0,05
Mnda	NM_001012029	-1,41	4,59	0,04
Was	NM_001108248	-1,41	3,88	0,04
Cytip	NM_001012086	-1,40	4,48	0,04
Snx20	NM_001024999	-1,40	3,20	0,05
Apbb1ip	NM_001100577	-1,39	4,08	0,05

Cadm1	NM_001012201	-1,39	3,41	0,05
Irf5	NM_001106586	-1,37	4,49	0,05
Naaa	NM_001010967	-1,37	3,66	0,05
Tbxas1	NM_012687	-1,37	3,66	0,05
Upb1	NM_053845	-1,37	6,33	0,04
Sptbn2	NM_019167	-1,36	5,12	0,04
S100a4	NM_012618	-1,36	4,53	0,05
Pcsk6	NM_012999	-1,35	4,15	0,05
Cxcr4	NM_022205	-1,34	4,31	0,05
Tgfbi	NM_053802	-1,33	4,54	0,05
Ddit4	NM_080906	-1,32	6,33	0,05
Mal	NM_012798	-1,32	6,03	0,05

Analysis was performed using https://reactome.org
